# PRELIMINARY ANALYSIS OF INTERLEUKIN-6 CHANGES IN PRE- AND POSTOPERATIVE IN DIABETIC PATIENTS WITH BMI<35 SUBMITTED TO PARTIAL DUODENAL SWITCH

**DOI:** 10.1590/0102-6720201600040009

**Published:** 2016

**Authors:** Luciano Dias de Oliveira REIS, Paulo Afonso Nunes NASSIF, Fernando Issamu TABUSHI, Fábio Quirillo MILLÉO, Giovani Marino FAVERO, Bruno Luiz ARIEDE, Cassiana Franco Dias Dos REIS, Bruno Franco DALABONA

**Affiliations:** Postgraduate Program in Principles of Surgery, Evangelic Faculty of Paraná/University Evangelic Hospital of Curitiba/Medical Research Institute, Curitiba, PR, Brazil

**Keywords:** Metabolic syndrome, Obesity, Diabetes mellitus type 2, Bariatric surgery, Interleukin-6.

## Abstract

**Background:**

Studies related to obesity have shown association with metabolic syndrome. Data showing that obesity is capable to cause low grade chronic inflammation, without its classic signs and symptoms, call attention to researches to study different cells types and the mechanism of the inflammatory process.

**Aim:**

To evaluate the variation of glycated hemoglobin (HbA1c) and the pro-inflammatory cytokine interleukin-6 (IL6) in diabetic patients with BMI <35 kg/m^2^ in the pre and postoperative of partial duodenal switch.

**Method:**

Nine patients were studied before and one year after the operation and the variation of the serum IL6 was measured by Elisa. The changes of HbA1c were also registered.

**Results:**

The pre-operative IL6 levels reached 65,50436±2,911993 pg/ml and one year after de operation 39,47739±3,410057 and the HbA1c average of 10,67 and 5.8 in the same period.

**Conclusion:**

The partial duodenal switch was efficient to control one year after the procedure the chronic inflammatory process caused by the diabetes mellitus type 2 with BMI <35 by dropping the IL6 levels and bringing the HbA1c to normal.

## RESUMO

**Racional:**

Os estudos relacionados à obesidade têm evidenciado sua associação com a síndrome metabólica. A descoberta que a obesidade é capaz de promover inflamação, sem os sinais clássicos, tem levado vários grupos de pesquisa a caracterizar os tipos celulares que agem e o mecanismo envolvido neste processo.

**Objetivo:**

Avaliar a variação da hemoglobina glicada e a secreção da citocina inflamatória, interleucina-6, em indivíduos diabéticos com IMC<35 kg/m² no pré e pós-operatório da técnica de desvio duodenal parcial.

**Métodos:**

Nove pacientes foram avaliados antes e um ano após a operação e a variação da concentração da interleucina-6 foi avaliada pela metodologia de Elisa. Também foi avaliada a variação da HbA1c.

**Resultados:**

A quantificação de interleucina-6 apresentou no pré-operatório valor de 65,50436±2,911993 pg/ml e de 39,47739+3,410057 pg/ml após um ano da operação e a hemoglobina glicada apresentou média de 10,67 no pré-operatório e de 5,8 após um ano da operação.

**Conclusão:**

O desvio duodenal parcial foi capaz de, um ano após o procedimento, diminuir os efeitos da inflamação crônica demonstrada pela diminuição da concentração da interleucina-6 plasmática e normalizar a hemoglobina glicada em pacientes diabéticos com IMC<35 kg/m^2^.

## INTRODUCTION

Medicine has been faced to astonished raise rate of obesity. Today, according to the WHO one billion person are overweight and tree hundred thousands are obese. 30% of the USA population has BMI>30^2^ ^,^ ^12^.

Studies related to obesity have shown wide association to insulin resistance, diabetes mellitus type 2 (DM2), hypertension, cardiovascular diseases, hypertriglyceridemia, the sum of these is known as metabolic syndrome (MS).

Data showing that obesity is capable to cause low grade chronic inflammation, without its classic signs and symptoms, call attention to researches to study different cells types and the mechanism of the inflammatory process.

The International Diabetes Federation indicates that the "obesogenic society" resulted from the sedentary habits associated to high caloric intake are responsible for the diabetic epidemic with impaired insulin function or low production on insulin by the pancreatic islet cells causing hyperglycemia and interacting with other lipid dysfunctions, oxidative stress and inflammatory response.

Cytokines are signaling polypeptides used in cellular communication by the immune system and in the inflammatory response, acting virtually in all types of cells and in the synthesis of RNAm. Among the cytokines, the IL6, object of this study, has been found in elevated levels in chronic diseases including DM2. IL6 mediated inflammation is implicated in age related disorders including atherosclerosis, peripheral vascular disease, coronary artery disease, dementia and Alzheimer 'disease, some forms of arthritis, cancer and DM2, the last one related to this paper.

Different surgical procedures upon obese subjects have been very efficient not only to reduce the BMI but also to improve the diseases linked to MS. Several publications are incisive in showing the benefits of bariatric procedures in DM2. These surgical procedures are also improving the inflammatory status of the patients allowing a new fied for researchers. Even diseases not related to MS, as asthma, have improved after the DM2 patients reached BMI below 30.

The objective of this study is to be the initial part of the evaluation of the anti-inflammatory effect achieved in diabetic patients with BMI below 35 kg/m² operated by the technique of partial duodenal diversion (PDS), analyzing the variation of levels of IL-6 and hemoglobin glucose levels in pre and one year postoperative diabetic patients with BMI<35.

## METHODS

This study has been approved by the Ethics Committee on the State University of Ponta Grossa under protocol 37/2010. All patients signed an informed consent

### Patients

Nine patients with BMI below 35, with DM2 at least for 2 years, with difficult controlled disease, were submitted to PDS. The surgical procedures were done at Vicentinio Hospital, in Ponta Grossa, PR, Brazil. All patients were diagnosed as DM2 according to WHO criteria. Patients with chronic infectious disease, cancer, pregnancy and drug or alcoholic addicts were excluded. Blood samples were obtained just before surgery, after 8 h fasting and one year after the operation, aiming to measure HbA1c and IL6.

### Surgical procedure

**FIGURE 1:**
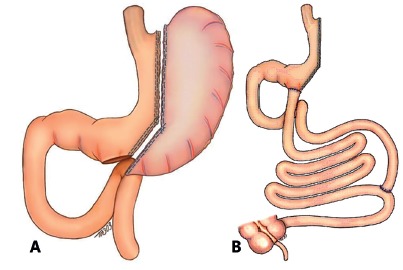
A) Vertical gastrectomy (sleeve); B) gastroentero anastomoses

Patients were admitted the day before surgery for clinical and endocronological evaluation. The operation was performed under general anesthesia. Laparotomy with a 10 cm midline incision was done. The greater omentun was separated from the transverse colon and removed. A vertical gastrectomy was started 6 cm proximal to the pylorus, after liberation of posterior adhesions and ligature with ultrasonic scalpel of the short gastric vessels. Using stapling devices, casted by Fouchet tube 32F, the vertical gastrectomy was progressive performed aiming the. The intestine was severed 260 cm from the duodenojejunal angle and the distal stoma sutured to the antrum (gastroileostomy). A distal anastomosis as performed at 80 cm from the ileocecal valve, end to side, in two planes, with the proximal severed small bowel to distal ileum. Both anastomoses were fashioned with poliglactin 000. The mesenteric spaces were closed (Figure 1).

### IL6 measurements 

An Elisa reader (Lab Life MX PN 2001) at 450 nm spectrum was used to measure IL6 using commercial Kit from eBioscience Inc. The reading was taking immediately after collection of the plasma samples and read at 450 wave length.

### HbA1c measurements

The level of HbA1c in blood samples were measured according to National Glycohemoglobin Standard Program using high performance liquid chromatographin

### Statistical analysis

Graf Pad Prism Stat version 4.0 program was used to evaluate the data and were considered significant for p<0.05. T student test was used to measure the IL6 levels.

## RESULTS

One of the nine patients was excluded due to the fact of having BMI just above 25. The others had BMI between 30-35 kg/m^2^. The BMI and laboratory data are shown at Table 1.

**TABLE 1:** Laboratory data and patients BMI

Pacients	BMI				% of BMI loss			Fasting glucose			Post prandial glucose			Triglycerides			Interleukin 6 levels		
	Pré	3m	6m	12m	Pre/3m	Pre/6m	Pre/12m	Pré	3m	6m	12m	Pré	3m	6m	12m	Pré	3m	6m	12m	Preoperative IL-6 (pg/mL)	Preoperative IL-6 (pg/mL)
1	53.47	22.40	55.55	55.55	15.40	14.90	14.90	385	128	94	96	410	172	145	153	200	112	57	74	-	-
2	33.27	27.83	22.38	22.80	16.40	32.70	31.50	235	103	114	100	234	161	154	160	186	134	97	117	56.48975	36.06728
3	32.15	26.13	24.17	24.31	18.70	24.80	24.40	220	97	90	94	379	153	138	148	370	160	85	78	61.59537	32.87626
4	29.69	24.87	24.45	25.03	16.20	17.60	15.70	273	124	89	86	342	143	155	150	259	110	85	73	58.72346	52.66054
5	30.56	28.20	28.20	28.43	7.70	7.7|	7	310	120	113	99	396	108	137	125	345	206	153	165	79.46503	39.58180
6	32.18	24.98	23.88	23.53	22.40	25.80	26.90	210	125	107	98	274	165	159	155	284	144	140	112	73.40211	38.30099
7	32.77	28.27	23.85	23.93	13.70	27.20	27	298	110	104	101	305	171	160	143	238	120	132	98	66.38189	37.98188
8	33.39	28.50	25.10	24.70	14.60	24.80	26	247	92	95	88	321	133	128	145	780	164	134	148	69.89200	39.12645
9	31.21	26.39	24.54	22.26	15.40	21.40	28.70	248	113.70	101	87	353	150	148	152	264	148	110	87	58.08525	39.09394
Mean	31.30	26.40	24.34	24.17	15.70	22.20	22.80	269.56	112.52	100.78	94.33	334.89	150.75	147.11	147.89	325.11	144.25	110.33	105.78	-	-
Standard deviation	2.19	2.05	1.70	1.87	6.40	22.40	14.80	54.72	12.96	9.40	5.89	-	-	-	-	-	-	-	-	-	-

The HbA1c analysis showed a progressive drop from the operation to one year after. The medium pre-operative level was 10.67 and after 12 months the medium level dropped to 5.88 (Table 2). 

**TABLE 2:** Means (%) of glycated hemoglobin in the different periods

Pacients	Pre	3m	6m	12m
1.	11,10	7,20	5,10	6,40
2.	8,30	6,80	7,10	6,70
3.	13,40	7,00	5,40	5,80
4.	10,50	6,50	5,40	5,10
5.	12,80	7,10	6, 20	6,50
6.	11,70	6,80	6,40	6,70
7.	10,30	6,70	6,30	6,10
8.	9,40	6, 20	5,10	4,80
9.	8,5	6,80	5,2	4,80
Mean	10,67	6,79	5,80	5,88
Standard deviation	1,78	0,31	0,72	0,79

**FIGURE 2:**
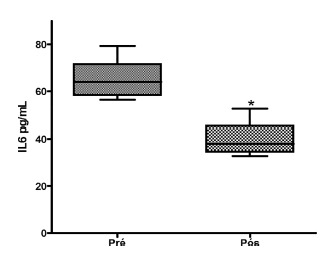
Evaluation of the plasma IL-6 quantification of pre and post-DSP (p <0.5)

The pre-operative IL6 levels reached 65.50436±2.911993 pg/ml and one year after de operation 39.47739±3.410057. T Student test showed significant decrease p<0.05 (Figure 2).

## DISCUSSION

Obesity and DM2 are very common in the modern society. Criteria for definition for both are well stabilized by the International Diabetes Federation and by the American Diabetes Association. Even though, the real mechanisms that conduct to the development of metabolic syndrome is unknown. Genetics and epigenetic factors in association to sedentary style of life and hyper alimentation are frequently mentioned in studies related do DM2 development. 

The capacity of fatty cells to in promoting insulin resistance makes the obesity an important risk factor to the development of DM2 even though the disease may occur in mild obese and may not in morbid obese patients.

The majority of reports on the effects of surgery in DM2 patients are in morbid obese patients since the criteria to bariatric/metabolic surgery has as main target the treatment of obesity. The most important criteria to choose patients to surgery are related to BMI and not the comorbities as hypertriglyceridemia, hypertension, and DM2. But the majority of DM2 patients are mild obese or obese grade I, since the pattern of these patients is abdominal obesity, excluding a great deal of DM2 patients with refratile disease as possible candidate to surgery.

In our study we are dealing with patients excluded from the classic protocol to bariatric surgery, since they obese I, with BMI below 35 or overweight, BMI >25 and <35, all with DM2 of difficult clinical control. Treating this group of not obese morbid patients the PDS caused a complete loss of excess of weight, with a drop of the BMI of 28% on average after one year.

Recent papers are suggesting to lower the BMI to treat patients with MS. According to the consensus of International Diabetes Federation, procedures to treat patients with MS may be indicated to patients with BMI of 35 or to patients between 30 and 35 if they have DM2 of difficult clinical control. In some Asian population with higher risks BMI of 27.5 is acceptable. In this study 8 (88%) patients were in the group with BMI between 30-35.

The results of metabolic surgery is consistently better in obese patients when compared to clinical treatment and has stimulated trials using different technics aiming the glycemic control in patients with BMI under 35, seeking to stablish surgery as an effective and definitive treatment to MS patients resistant to clinical treatment, a commoner growing problem in worldwide population.

Most of the surgical bariatric procedures used today were described more than 20 years ago and designed to treat morbid obese patients before the knowledge of the entero-hormones. Santoro et all published and staged evolutive adaptative procedure to treat MS patients and from his group arouse the concept of the operation used in this study, PDS. This procedure innovates not excluding any segment of stomach or bowel, allowing a rapid transit of the alimentary bolus without dumping or iron or calcium deficiency and an early stimulus to distal ileum.

In this study, the results of PDS in DM2 patients were very satisfactory. From the 9 patients in this group, six were on daily use of insulin and one refused to use insulin despite of his endocrinologist recommendation. The indication of insulin was suspended in all patients after the operation and the patients were orientated to measure regular fasting glucose levels and return if it reached >200 ml/dl. None of these patients need to return to insulin during the first year. Only one patient was kept on oral hypoglycemic drug (a DPP IV inhibitor) according to the endocrinologist to improve the MS, despite of the HbA1c below 7 mg%. The average of pre-operative HbA1c was 10.67% . A significant drop of the HbA1c (p<0.05) follow de operation, below 7 in the first trimester and and reaching an average of 5.88% one year after the surgery. The average fasting glycemic level before PDS was 269 mg% and 94.3 mg% one year post-operative, considered very acceptable.

Cohen at al. reported 37 patients submitted to laparoscopic gastric bypass (RYGB) and De Paula et al. published the results with sleeve gastrectomy and ileal interposition (ileal break) in 30 patients. Both studies were in patients with BMI under 35 kg/m^2^ and reached diabetes control level s above 90% of patients.

When RYGB is used the segment of small bowel in contact with the ingested food is the jejunum, being the results not as expressive as when biliopancreatic bypass with duodenal switch is used, when the ileum is in contact to food. It has been suggested that the size of proximal excluded segment (biliopancreatic loop) is very important in the antidiabetogenic final result of the procedure. Valezi reported in a prospective study in obese patients submitted to RYGB that the size of the alimentary or biliopancreatic loop did not interfere with the weight loss of their patients. PDS do not only interfere in the amount of food ingested due to the vertical gastrectomy but also places the ileum in contact to the alimentary bolus via gastroileostomy improving the release of incretins, mainly GLP-1. In our study 88% of patients reached DM2 control without exclusion of any gastrointestinal segment.

Aiming to set up a pattern, Nassif et al. in 2013 described a technique for vertical gastrectomy placing a Fouchet's tube 32 F in the lesser curvature to cast the neo stomach, starting stapling the greater curvature just proximal to the pylorus, creating a narrow and long stomach. In 55 patients submitted to vertical gastrectomy, Nassif obtained control of 84.6% of the DM2 in the first year and 91.6% in the second year. In a long revision of 27 reports, when vertical gastrectomy was used as the only procedure, an average of 66% remission rate for the DM2 patients was obtained. 

Early publication of this group of researches using PDS has shown, using small metal radiopaque spheres mixted to a meal, that 40% of ingested contend exited through the pylorus to duodenum and 60% via gastroileostomy. Keeping the duodenum in the PDS is responsible for avoiding lacking of absorption of important substances, essentials to human metabolism. Chronic anemia is common after RYGB and demands oral or parenteral iron replacement. Keeping the pylorus intact also avoids dumping syndrome, a frequent complication of gastric operations. In our patients submitted to PDS, none had nutritional deficits or dumping and after one year of the procedure the median hematocrit was 39 mg/dl. 

The duodenum and proximal jejunum is rich in K cell, responsible for the production of GIP, suggesting that the duodenum is important in controlling glycemic levels (foregut hypothesis) linked to this incretinic enterohormone action. The greater capacity of distal small bowel, rich in GLP 1 producing L cells, is also very important in regulating glycemic levels (hindgut hypothesis) after metabolic procedures.

Visceral fat, mainly the omentum, is responsible to secrete resistin, a peptide that acts on miocites, liver and even on the adipocites, impairing insulin sensitivity and inducing to DM2. The metabolic operation associated to resection of a large part of the omentum also results in lesser secretion of PAI-1 and decrease cardiovascular risks and improves lipid profile.

In this study we also measured the fasting triglycerides before and one year after the PDS. There was a significant drop in triglycerides (p<0.05). The pre-operative median levels were 325.11 mg/dl and one year after the operation only 105.78 mg/dl, considered normal. Santoro comparing two surgical techniques - RYGB and gastroentero omentectomy - found a significant improvement (p<0.05) in post-prandial triglycerides.

DM2 and obesity are diseases linked to low grade chronic inflammatory activity, resulting in impaired insulin sensibility and insulin resistance. Low grade chronic systemic inflammation is so called when the inflammatory citokines are two to three times higher than normal levels and generally there is a parallel increase in protein C reactive.

Obesity is considered a risk factor to insulin resistence. The adipose tissue is considered a endocrine organ since it produces several molecules, among them, adiponectin, leptin, resistin, TNF alfa, Interleukins. The interleukin 6 (IL6), a pro-inflammatory cytokine, was objective of this study.

Omoigui in 2007 related that the effective treatment of all diseases related to low grade chronic inflammatory activity should aim to lower or inhibit IL6. The beneficial effects of a diet rich in polyfhenols found in plants, fruits, cereals, vegetables, chocolate and drinks like wine (resveratrol) contributes to lower the IL6 levels and helps on inhibiting the chronic inflammatory activity. Some minerals, including the anti-oxidant selenium, also improve the cellular response to inflammation and homeostasis.

IL6 may also acts as an anti-inflammatory cytokine, when produced in the skeletal muscle, and then is known as myokin, giving to muscles a status of "endocrine organ". It is released early during exercises, improving muscular insulin sensibility.

In a recent report from Denmark, a group of 25 patients were submitted to RYGB and after one week of the procedure a sharp rise in plasma IL6 was found possible related to surgical trauma, but a progressive drop in IL6 was found at three and 12 months postoperative. In the same study, the authors reported an improvement of others inflammatory parameters. As in this study a one year decrease in IL6 was found. 

Buchwald in two metanalysis reported that how much more disabsortive are the procedures bigger is the weight loss and higher is the DM2 resolution. In the first report, in 2004, 1846 diabetic patients were included. The DM2 resolution rate was 98% in the biliopancreatic group, 83% in RYGB group and in the adjustable gastric band 47.9%. The second report, in 2009 confirmed the previous findings.

In our review we did not find any study using PDS for the treatment of DM2 patients with BMI below 35, neither measuring IL6 as parameter for DM2 low rate chronic inflammatory resolution. The PDS controlled the MS and induced DM2 remission parallel to IL6 drop to normal. This procedure is technically simple, safe, and efficient in controlling the DM2 and excess of weight, without iron and calcium deficiency by keeping the duodenum in the alimentary transit. 

According to Santoro et al., the PDS, referred by him as vertical gastrectomy with transit bipartition, has been designed to work primarily through metabolic ways, avoiding restriction and malabsorption. Absence of prosthesis or excluded segment, full endoscopic access, and easy feasibility associated with a metabolic corrective intervention bring benefits to patients. PDS is simple, reversible, and improves the results of vertical gastrectomy (with is the commonest bariatric-metabolic procedure performed in USA since 2015). We believe that PDS is one more step toward the excellence of metabolic surgery.

## CONCLUSION

The partial duodenal switch was efficient to control one year after the procedure the chronic inflammatory process caused by the DM 2 with BMI <35 by dropping the IL6 levels and bringing the HbA1c to normal.
